# Distribution of Zinc in Mycelial Cells and Antioxidant and Anti-Inflammatory Activities of Mycelia Zinc Polysaccharides from *Thelephora ganbajun* TG-01

**DOI:** 10.1155/2020/2308017

**Published:** 2020-06-17

**Authors:** Lan Zheng, Yaohong Ma, Yunjuan Zhang, Qingjun Meng, Junhui Yang, Weili Gong, Qingai Liu, Lei Cai, Lujiang Hao, Binglian Wang, Yan Yang

**Affiliations:** ^1^Biology Institute, Qilu University of Technology (Shandong Academy of Sciences), Jinan 250103, China; ^2^Shandong Provincial Key Laboratory of Biosensors, Jinan 250103, China; ^3^State Key Laboratory of Biobased Material and Green Papermaking, Qilu University of Technology (Shandong Academy of Sciences), Jinan 250353, China

## Abstract

This study demonstrates that *Thelephora ganbajun* had a strong ability to absorb zinc, and zinc can be compartmentally stored in the small vesicles and mainly accumulated in the form of zinc-enriched polysaccharides (zinc content was 25.0 ± 1.27 mg/g). Mycelia zinc polysaccharides (MZPS) and its fractions were isolated. The main fraction (MZPS-2) with the highest antioxidant activity *in vitro* was composed of mannose : galacturonic acid : glucose : galactose in a molar ratio of 61.19 : 1 : 39.67 : 48.67, with a weight-averaged molecular weight of 5.118 × 10^5^ Da. MZPS-2 had both *α*-pyranose and *β*-pyranose configuration and had a triple helical conformation. By establishing zebrafish models, we found that MZPS-2 can significantly scavenge free radicals, reduce the generation of reactive oxygen species caused by inflammation, and inhibit the recruitment of neutrophils toward the injury site. Therefore, MZPS-2 exhibited antioxidant and anti-inflammatory effects and can be used as a zinc supplement with specific biological activities to alleviate zinc deficiency complications, such as chronic oxidative stress or inflammation.

## 1. Introduction

Nutritional imbalance caused by zinc deficiency is one of the major public health concerns worldwide [1]. Epidemiological studies have shown that at least one-fifth of the world's population is at risk of inadequate zinc intake, and the true prevalence of marginal zinc deficiency is unknown because of the nonspecific nature of symptoms [2]. Zinc, the second most abundant trace element in the body, is required for numerous biological processes [3]. Zinc deficiency can lead to various abnormalities; chronic oxidative stress and inflammation are the most common complications of zinc deficiency [4–6].

Due to the important physiological role of zinc, studies have aimed to find a safe and effective source of zinc supplement. With the continuous increase in the health consciousness of the public, people are increasingly hoping to replace chemically synthesized organozinc supplements with natural organozinc supplements. The most suitable approach for the preparation of natural organozinc is biotransformation [7]. Edible fungi have a stronger ability to absorb and transform metal elements, and edible fungi can transform inorganic zinc in the environment to bioorganic zinc in cells [8–10]. The bioorganic zinc in edible fungus cells has high bioavailability and low toxic side effects. The biotransformation of zinc by edible fungi has gradually attracted attention, but the mechanism of biotransformation remains unknown.

*Thelephora ganbajun* is a rare wild edible fungus that is distributed in pine forests in high-altitude areas of Yunnan Province, southwestern China [11–15]. In the present study, we found that *T. ganbajun* could accumulate zinc in the form of zinc-enriched polysaccharides. Zinc polysaccharides are an ideal source of zinc supplement, and they have the biological effects of fungal polysaccharides. Edible fungus polysaccharides are high-molecular-weight polymers isolated from fungal mycelia, fruiting bodies, and fermentation broth, which have been demonstrated to have a wide range of biological activities such as antioxidation, anti-inflammation, immunomodulation, and antitumor activities [16–20]. However, to our knowledge, there have been few reports focusing on the structural feature and activity of *T. ganbajun* polysaccharides.

The aim of this study was to develop a new organic zinc supplement with specific biological functions through liquid fermentation of *T. ganbajun*. Therefore, we analyzed the zinc absorption capability of *T. ganbajun* and determined the distribution and accumulation of zinc in mycelial cells *in situ*. In addition, *T. ganbajun* mycelia zinc polysaccharides (MZPS) were successfully isolated. Three major fractions of MZPS (MZPS-1, MZPS-2, and MZPS-3) were selected to investigate the relationship between chemical characterization and antioxidant activity. We also explored the antioxidant and anti-inflammatory activities of MZPS-2 *in vivo* by establishing zebrafish models. This study suggested that organification of zinc through edible fungus liquid fermentation provided a novel method to produce a zinc supplement.

## 2. Materials and Methods

### 2.1. Chemicals

The monosaccharide standards (D-mannose, D-rhamnose, glucuronic acid, galacturonic acid, D-glucose, L-arabinose, and L-galactose), 1-phenyl-3-methyl-5-pyrazolone (PMP), 2, 2-diphenyl-1-picryl-hydrazyl (DPPH), 2, 2′-azino-bis (3-ethylbenzothiazoline-6-sulfonic acid) (ABTS), and lipopolysaccharide (LPS) were purchased from Sigma-Aldrich Co. (St. Louis, MO, USA). Zinquin ethyl ester was purchased from Biotium, Inc. (Fremont, CA, USA). DEAE-52 was purchased from Whatman Co. (UK). Sephadex-G 100 was purchased from Solarbio Co. (Beijing, China). Zn Standard Solution (99.999%) was purchased from SPEX CertiPrep Co. (USA). The diagnostic kit for reactive oxygen species (ROS) was purchased from Nanjing Jiancheng Bioengineering Institute (Nanjing, China). All other chemicals were of analytical grade from local chemical suppliers in China.

### 2.2. Strain and Culture

*T. ganbajun* strain TG-01 used in this experiment was provided by our laboratory (Jinan, China). Liquid fermentation technology was used to produce zinc-enriched mycelia. *T. ganbajun* TG-01 was grown in 500 mL Erlenmeyer flasks containing 250 mL of liquid medium (200 g/L fresh potato, 20 g/L dextrose, 1 g/L MgSO_4_·7H_2_O, 1 g/L KH_2_PO_4_, and 300 mg/L zinc). Zinc was added in the form of ZnSO_4_·7H_2_O, and the liquid medium of the non-zinc-enriched mycelia did not contain ZnSO_4_·7H_2_O. The liquid culture was grown at 25°C with shaking at 130 r/min for 7 days.

### 2.3. Determination of Zinc Enrichment Capability.

*T. ganbajun* TG-01 was cultured in liquid medium with different zinc concentrations (0, 50, 100, 150, 200, 300, 400, 500, 600, 700, and 800 mg/L). After 7 days of cultivation, the mycelia were collected by centrifugation, washed, dried, weighed, and pulverized. A 0.1 g sample was mixed with 10 mL of HNO_3_ and 3 mL of HClO_4_, and the mixture was stored overnight. Then, the mixture was boiled until the solution was clarified and subsequently diluted to 50 mL with deionized water. The zinc content in the solutions was determined by inductively coupled plasma-atomic emission spectrometer (ICP-AES) (IRIS Advantage OPTIMA 7000DV, Thermo Fisher Scientific, Rockford, IL, USA) (1150 W RF power, 0.5 L/min auxiliary flow, 0.7 L/min nebulizer flow, and 50 r/min pump rate).

### 2.4. Fluorescence Detection of Zinc Distribution

*T. ganbajun* TG-01 was cultured in liquid medium with added zinc (300 or 600 mg/L). The zinc-enriched mycelia were collected by centrifugation (10,000 × *g*, 10 min, 4°C) and washed three times with a solution of MgSO_4_·7H_2_O (0.6 mol/L). Mycelia were incubated with shaking (150 r/min, 1 h, 25°C) in the MgSO_4_·7H_2_O solution (0.6 mol/L) containing zinquin ethyl ester (Biotium, Inc., Fremont, CA, USA) (1 *α*g/mL), a zinc fluorescent marker. After the incubation, mycelia were washed four times with MgSO_4_·7H_2_O solution (0.6 mol/L). The fluorescently stained mycelia were mounted on glass slides and viewed with a laser scanning confocal microscope (LSCM) (Olympus FV1200, Japan).

### 2.5. Determination of Zinc Enrichment Ratio of Cellular Components

Cellular components (polysaccharides, proteins, and nucleic acids) in the zinc-enriched mycelia were separately extracted. Polysaccharides were extracted following the water extraction and alcohol precipitation method [21]. The mycelium powder was added to deionized water at a ratio of 1 : 20, sonicated (300 W, 10 min), and then incubated at 90°C for 2 h. The process was repeated three times. After centrifugation (10,000 × *g*, 10 min, 4°C), the combined supernatant was concentrated to one third of the initial volume using a rotary evaporator (CCA-1112A, Eyela, Japan) at 50°C and precipitated with a threefold volume of anhydrous ethanol for 12 h. The precipitate was collected by centrifugation (10,000 × *g*, 10 min, 4°C) and dried at 55°C. The crude polysaccharides were redissolved in deionized water, deproteinized with Sevag reagent (chloroform/n-butanol, 5 : 1 *v*/*v*) and lyophilized.

Nucleic acids were extracted by the high-concentration salt method [22] with slight modifications. The zinc-enriched mycelia powder (8 g) was mixed with 250 mL of NaCl solution (10%), and the mixture was incubated at 95°C for 1 h. After centrifugation (10,000 × *g*, 10 min, 4°C), the supernatant was deproteinized twice following the Sevag method, and then, the pH was adjusted to 2–2.5 with HCl solution. The resulting solution was incubated in an ice-water bath for 30 min and centrifuged (10,000 × *g*, 10 min, 4°C). The precipitate was washed three times with 95% ethanol and dried at 55°C.

Proteins were extracted following the dilute alkali method [23] with slight modification. The zinc-enriched mycelia powder (4 g) was mixed with 120 mL of NaOH solution (0.25 mol/L), and the mixture was incubated at 50°C for 4 h and centrifuged (10,000 × *g*, 10 min, 4°C). Then, (NH_4_)_2_SO_4_ was added to the supernatant to 95% saturation and it was incubated overnight at 4°C. The precipitate was collected by centrifugation (6,000 × *g*, 10 min, 4°C), washed three times with ethanol-diethyl ether (2 : 1 *v*/*v*) mixture and then dried at 55°C.

The prepared nucleic acid, polysaccharide, and protein components were weighed, and the zinc content was determined from each component. The following formula was used calculate the zinc enrichment ratio of each cellular component separately: zinc enrichment ratio (%) = zinc content of each component (mg/g) × yield (g/g)/zinc content of mycelia powder (mg/g). The yield was calculated as follows: yield (mg/g) = weight of each component (mg)/weight of mycelia powder (g).

### 2.6. Isolation and Purification of MZPS

The MZPS and mycelia polysaccharides (MPS) were separately extracted from zinc-enriched and non-zinc-enriched mycelia following the water extraction and alcohol precipitation method. MZPS and MPS was dissolved in deionized water and filtered, then loaded onto a DEAE-52 cellulose column (1.6 cm × 30 cm, Whatman, UK). The column was stepwise eluted with different concentrations of NaCl solution (0, 0.05, 0.1, and 0.3 mol/L) at a flow rate of 1.0 mL/min. The eluted fractions (3 mL/tube) were collected automatically and detected by the phenol sulfuric acid method [24]. The main fractions were further purified using a Sephadex-G 100 column (1.6 × 50 cm, Solarbio Co., Beijing, China) and eluted with deionized water at a flow rate of 0.1 mL/min. Each fraction was collected and lyophilized for further research. The total sugar content was determined by the phenol sulfuric acid method.

### 2.7. Characterization Analysis

#### 2.7.1. Fourier Transform Infrared Spectroscopy (FT-IR) Analysis

An FT-IR analysis of the MZPS and its fractions were measured following the potassium bromide (KBr) pellet method. The FT-IR spectra were recorded using a Thermo-Nicolet 6700 FTIR spectrophotometer (Thermo Scientific, Rockford, IL, USA) with OMNIC software (Version 8.2, Thermo Scientific, Rockford, IL, USA) in the range of 4,000–400 cm^−1^.

#### 2.7.2. Determination of the Monosaccharide Composition

The MZPS and its fractions (50 mg) were hydrolyzed with 10 mL of H_2_SO_4_ (1 mol/L) in a sealed ampoule filled with N_2_ at 100°C for 10 h. The excess acid was neutralized using NaOH (2 mol/L). The resulting solutions were analyzed to obtain monosaccharide components using high-performance liquid chromatography (HPLC) after PMP precolumn derivation according to the procedure of Zheng et al. [21].

#### 2.7.3. Determination of Molecular Weight

The molecular weight of polysaccharides was determined by high-performance liquid gel permeation chromatography (HPGPC) that was operated with an 18-angle laser light scattering high gel permeation chromatography system equipped with a Shodex SBOHPAK-806-803 column (8 mm × 300 mm, Showa Denko K.K., Tokyo, Japan), a GPC gel permeation chromatograph unit infusion pump (Waters 515, Waters Co., Milford, MA, USA), an 18-angle laser light scattering (Wyatt Dawn Heleos11, Wyatt Technology Co., Santa Barbara, CA, USA), and a refractive index detector (Optilab T-rex, Wyatt Technology Co., Santa Barbara, CA, USA). The column was eluted with double-distilled water at a flow rate of 1 mL/min. Dextrans were used as standards. The molecular weight was analyzed using Astra software (Version 5.3.4, Wyatt Technology Co., Santa Barbara, CA, USA).

#### 2.7.4. Determination of Triple Helical Structure

The triple-helix structure of MZPS and its fractions were determined via the Congo red method [25]. Aliquots of sample solution (1 mL, 6 mg/mL) were mixed with 1 mL Congo red solution (80 *μ*mol/L). The mixture was gradually adjusted to different NaOH concentrations (0–1 mol/L) by adding 1 mL of the different concentrations of NaOH solution (0–3 mol/L). At each NaOH concentration, the maximum absorption wavelength (*λ*_max_) was measured using a microplate spectrophotometer (Dynex Spectra MR, Dynex Technologies, Chantilly, VA, USA) in the 300–700 nm range after allowing the sample to equilibrate for 5 min.

### 2.8. Determination of Antioxidant Activities In Vitro

#### 2.8.1. Hydroxyl Radical Scavenging Assay

The hydroxyl radical scavenging activity was determined by the Fenton reaction [26]. Briefly, MZPS and its fractions were diluted in a series of concentrations using deionized water. The reaction mixture contained 1 mL of ferrous sulfate solution (9 mmol/L), 1 mL of salicylic acid ethanolic solution (9 mmol/L), 1 mL of polysaccharide sample solution, and 1 mL of hydrogen peroxide solution (8.8 mmol/L). After rapid shaking, the mixture was incubated at 37°C for 30 min and then centrifuged at 5,000 r/min for 10 min. The absorbance of the supernatant at 510 nm was measured. Vitamin C was used as a positive control. The hydroxyl radical scavenging rate (%) = [(*A*_0_ − *A*)/*A*_0_] × 100, where *A* is the absorbance of the sample solution and *A*_0_ is the absorbance of the blank (deionized water instead of the sample).

#### 2.8.2. DPPH Radical Scavenging Assay

The DPPH radical scavenging activity was measured according to the reported method [27]. In brief, 2 mL of polysaccharide sample solution at various concentrations was mixed with 2 mL of DPPH ethanol solution (0.2 mmol/L). The mixture was kept in the dark for 30 min; then, the absorbance was determined at 517 nm and BHT was used as the control. The DPPH scavenging rate (%) = [1 − (*A* − *A*_0_)/*A*_1_] × 100, where *A* is the absorbance of the sample solution, *A*_0_ is the absorbance of the background (ethanol instead of DPPH), and *A*_1_ is the absorbance of the blank (deionized water instead of the sample).

#### 2.8.3. ABTS Radical Scavenging Assay

The ABTS radical scavenging activities were measured as previously described [28]. The ABTS solution (7 mmol/L) and potassium persulfate solution (4.9 mmol/L) were mixed in an equal volume and stored in the dark 16 h. The mixture was diluted with PBS to an absorbance of 0.70 ± 0.02 at 734 nm. For polysaccharide samples, 1 mL was mixed with 3 mL of the diluted ABTS solution and stored in the dark for 6 min. Then, the absorbance was measured at 734 nm and BHT was used as the control. The ABTS scavenging rate (%) = [1 − (*A* − *A*_0_)/*A*_1_] × 100, where *A* is the absorbance of the sample solution, *A*_0_ is the absorbance of background (PBS instead of ABTS solution), and *A*_1_ is the absorbance of the blank (deionized water instead of the sample).

### 2.9. Determination of Antioxidant and Anti-Inflammatory Activities In Vivo

#### 2.9.1. Zebrafish Maintenance and Embryo Collection

Wild type (AB strain) and transgenic strains (Tg(krt4:NTR-hKitGR)cy17 and Tg(Lyz:GFP)) of zebrafish (*Danio rerio*) were obtained from our laboratory's Zebrafish Drug Screening Platform (Jinan, China). Zebrafish were kept at 28°C with a 14 : 10 hr light : dark cycle in an aquarium with standard fish water (5 mmol/L NaCl, 0.17 mmol/L KCl, 0.4 mmol/L CaCl_2_, and 0.16 mmol/L MgSO_4_) [29–31]. At 4 hours post fertilization (hpf), the embryos of transgenic strains were examined under an Olympus SZX16 stereo microscope (Olympus, Tokyo, Japan), and normally developing and fluorescent embryos were selected for subsequent experiments. Zebrafish larvae were not fed during the first 7 days post fertilization (dpf). All tests were carried out in triplicate and complied with the Regulation on the Administration of Laboratory Animals (2013 Revision, Document Number: Order No. 638 of the State Council). The animal protocols used in this work were evaluated and approved by the Laboratory Animal Welfare and Ethic Committee of the Biology Institute (Protocol 2018 No. 62, 26 September 2018, Jinan, China).

#### 2.9.2. Determination of Antioxidant Activity of MZPS-2

The Tg(krt4:NTR-hKitGR)cy17 strain of zebrafish embryos was collected at 24 hpf, and the egg membranes were removed by 1 mg/mL proteases (Pronase E, Solarbio Co., Beijing, China). Then, zebrafish larvae were transferred into 24-well cell culture plates (10 larvae per well in 2 mL of solution) and randomly divided into following exposure groups. Vehicle control groups (VC) were exposed to fish water. Model control groups (MC) were exposed to fish water with metronidazole (10 mmol/L). Positive control groups (PC) were exposed to fish water with vitamin C (25 *μ*g/mL) and metronidazole (10 mmol/L). Three treated MZPS-2 groups (I, II, and III) were exposed to fish water with MZPS-2 (10, 25, or 50 *μ*g/mL, respectively) and metronidazole (10 mmol/L). Three treated MPS-2 groups (IV, V, and VI) were exposed to fish water with MPS-2 (10, 25, or 50 *μ*g/mL, respectively) and metronidazole (10 mmol/L). All groups were incubated for 24 hours until 48 hpf at 28°C. After anesthetizing with 0.16% tricaine, the entire lateral view of the larvae was imaged using an Olympus SZX16 stereomicroscope equipped with an Olympus DP72 camera (Olympus, Tokyo, Japan). The number of fluorescent points was determined for the trunk area using Image-Pro Plus software (Media Cybernetics, Bethesda, MD, USA).

#### 2.9.3. Inhibition of Inflammation-Induced Intracellular ROS Generation

The zebrafish larvae at 72 hpf were distributed into 24-well cell culture plates (10 larvae/well in 2 mL of solution) and randomly divided into following exposure treatments: vehicle control treatment (VC), in which zebrafish larvae were treated with fish water; model control treatment (MC), where larvae were treated with fish water containing 25 *μ*g/mL LPS; the MZPS-2 treatments (I, II, and III), where larvae were treated with fish water containing 25 *μ*g/mL LPS and MZPS-2 (10, 25, or 50 *μ*g/mL, respectively); and the MPS-2 treatments (IV, V, and VI), where larvae were treated with fish water containing 25 *μ*g/mL LPS and MPS-2 (10, 25, or 50 *μ*g/mL, respectively). After incubating at 28°C for 72 h, larvae were treated with 20 *μ*g/mL DCF-DA for 40 min, followed by gentle washing. Larvae were anesthetized with 0.16% tricaine, and the stained embryos were imaged using an Olympus SZX16 stereomicroscope equipped with an Olympus DP72 camera (Olympus, Tokyo, Japan). Fluorescence intensity was quantified using ImageJ 1.46r software (Wayne Rasband, National Institutes of Health, Bethesda, MD, USA).

#### 2.9.4. Inhibition of Inflammation-Induced Neutrophil Migration

To evaluate the effects of MZPS-2 on neutrophil migration, Tg(Lyz:GFP) transgenic zebrafish larvae at 72 hpf were distributed into 24-well cell culture plates (10 larvae/well in 2 mL of solution). Zebrafish larvae were exposed to MZPS-2 at the doses of 10, 25, or 50 *μ*g/mL (I, II, and III, respectively), MPS-2 at the doses of 10, 25, or 50 *μ*g/mL (IV, V, and VI, respectively), or indomethacin at a dose of 5 *μ*g/mL (PC) at 28°C for 2 h. Then, larvae were exposed to 20 *μ*mol/L CuSO_4_ for 2 h to stimulate inflammation-induced neutrophil migration. Zebrafish treated with fish water were used as vehicle controls (VC), and zebrafish treated with 20 *μ*mol/L CuSO_4_ were used as model controls (MC). All larvae were treated with 4% paraformaldehyde for 1 h, and then, larvae were rinsed with phosphate buffered solution. After anesthetizing with 0.16% tricaine, images were obtained using an AXIO ZOOM.V16 stereomicroscope equipped with an Axiocam 506 Color camera (ZEISS, Oberkohen, Germany), and the number of neutrophil migration was measured from these images using Image-Pro Plus software (Media Cybernetics, Bethesda, MD, USA).

### 2.10. Statistical Analysis

All data were expressed as mean ± standard deviation (SD) from at least three independent experiments. Comparisons among experimental groups were performed using a one-way analysis of variance (ANOVA). SPSS version 18.0 software (SPSS, Inc., Chicago, IL, USA) was used for statistical analysis. *P* < 0.05 was considered statistically significant.

## 3. Results and Discussion

### 3.1. Analysis of Zinc Enrichment Capability

**Figure 1:**
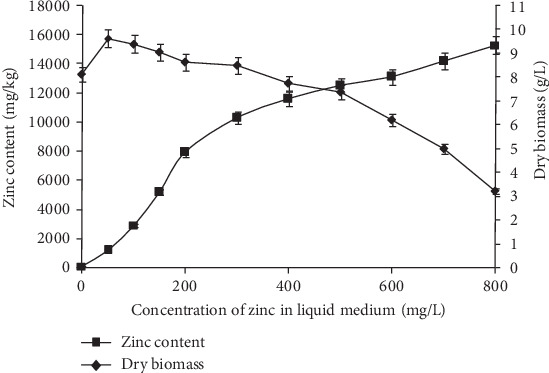
Changes in zinc content and dry biomass. The zinc content was determined by inductively coupled plasma-atomic emission spectrometer. Data are presented as means ± SD (*n* = 3).

As shown in Figure 1, when the zinc concentration in the liquid medium was less than or equal to 300 mg/L, *T. ganbajun* mycelia showed improved or normal growth. When the zinc concentration was greater than 300 mg/L, the zinc gradually inhibited the growth of the mycelia and the dry biomass rapidly decreased. Thus, *T. ganbajun* exposed to a concentration of 300 mg/L or less zinc showed homeostasis, whereas a concentration greater than 300 mg/L zinc caused poisoning conditions that negatively affected mycelial growth.

At 0 mg/L, the zinc content was 52 ± 2.6 mg/kg in dry mycelia, and the dry biomass was 8.12 ± 0.31 g/L (the basal zinc content in 0 mg/L liquid medium was about 1.3 mg/L). In order to obtain zinc-enriched mycelia at a reasonable production rate, a zinc concentration of 300 mg/L in liquid medium was chosen to produce MZPS. At 300 mg/L, the zinc content was 10.3 ± 0.42 mg/g in dry mycelia, and the dry biomass was 8.50 ± 0.33 g/L. In addition, the zinc content was 0.112 ± 0.003, 0.105 ± 0.003, 25.0 ± 1.27, and 25.9 ± 1.33 mg/g in MPS, MPS-2 (the main fraction of MPS), MZPS, and MZPS-2 (the main fraction of MZPS), respectively. Therefore, the zinc content of MZPS and MZPS-2 was significantly higher than that of MPS (*P* < 0.01) and MPS-2 (*P* < 0.01), respectively.

### 3.2. Analysis of Zinc Distribution in Cells

**Figure 2:**
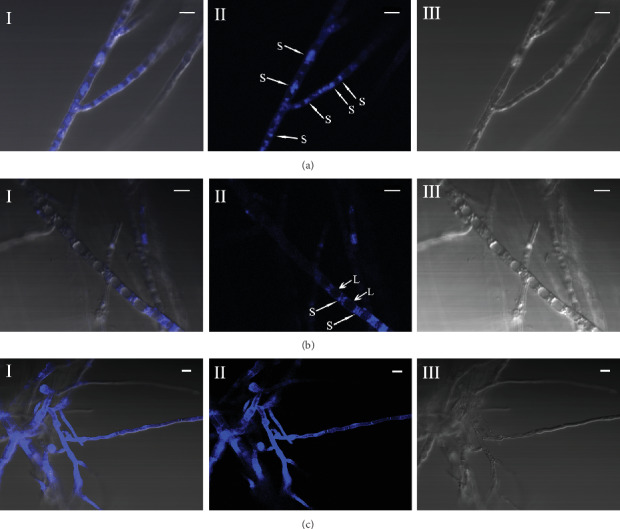
Fluorescence microscopy images showing zinc distribution in *T. ganbajun* cells. (a) The zinc concentration was 300 mg/L, and the duration of the culture was 7 d. (b) The zinc concentration was 300 mg/L, and the duration of the culture was 11 d. (c) The zinc concentration was 600 mg/L, and the duration of the culture was 7 d. I: II and III overlay images; II: fluorescent images; III: black and white images. Pictures represent more than 70% of fluorescence microscopy images (number of repeats = 30). Zinc is labeled by a zinc fluorescent probe (zinquin ethyl ester). S: small vesicle; L: large vacuole. Scale bars = 5 *μ*m.

Fluorescence microscopy images showing zinc distribution in *T. ganbajun* TG-01 mycelia exposed to concentrations of 300 or 600 mg/L zinc, and these two representative zinc concentrations provided either homeostasis or poisoning conditions for mycelia growth, respectively. As shown in Figure 2(a), when mycelia were grown under homeostasis conditions, the zinc fluorescent probe (zinquin ethyl ester, Biotium, Inc., Fremont, CA, USA) labeled numerous small vesicles in mycelia, indicating that cytosolic compartmentalization of zinc has occurred. As shown in Figure 2(b), as the duration of the culture increases, large vacuoles form in the aged mycelia, and the zinc did not accumulate in these large vacuolar compartments; zinc accumulation was still evident in small cytoplasmic vesicles. As shown in Figure 2(c), when the zinc concentration was 600 mg/L, the branching of hyphae increased and all of the hyphae fluoresced blue, indicating that the cytosolic compartmentalization of zinc did not occur under poisoning conditions. The cytosolic compartmentalization of zinc can avoid the poisoning caused by free zinc, which may be a reason why edible fungi have strong zinc endurance and absorption ability.

### 3.3. Analysis of Zinc Enrichment Ratio of Cellular Components

The zinc enrichment ratio of cellular components (nucleic acids, proteins, and polysaccharides) is presented in Table 1. The zinc content and zinc enrichment ratio of polysaccharides were significantly higher (*P* < 0.01) than those of the proteins and nucleic acids, indicating that zinc is more readily accumulated in polysaccharides.

### 3.4. Isolation and Purification of MZPS

**Figure 3:**
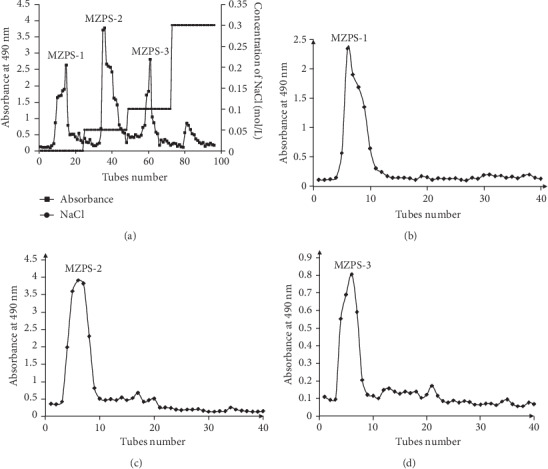
(a) Elution curve of MZPS on a DEAE-52 column. Elution curves of (b) MZPS-1, (c) MZPS-2, and (d) MZPS-3 on a Sephadex-G 100 column.

As shown in Figure 3(a), four fractions (MZPS-1, MZPS-2, MZPS-3, and MZPS-4) were sequentially eluted using 0, 0.05, 0.1, and 0.3 mol/L NaCl by DEAE-52 cellulose anion exchange chromatography. The yields of MZPS-1, MZPS-2, MZPS-3, and MZPS-4 from the total MZPS were 139.56 ± 8.62, 611.64 ± 42.78, 119.17 ± 9.15, and 66.12 ± 3.93 mg/g, respectively. As shown in Figures 3(b), 3(c), and 3(d), three major fractions (MZPS-1, MZPS-2, and MZPS-3) were shown as a signal and symmetrical peak in their respective Sephadex-G 100 chromatograms, indicating that each fraction contained a relatively homogeneous polysaccharide. Moreover, the total sugar content in MZPS, MZPS-1, MZPS-2, and MZPS-3 were 882.76 ± 38.7, 901.32 ± 42.12, 922.75 ± 43.58, and 895.61 ± 32.96 mg/g, respectively. In addition, MPS and its fractions (MPS-1, MPS-2, and MPS-3) showed similar elution curves. The total sugar content in MPS, MPS-1, MPS-2, and MPS-3 were 875.27 ± 36.4, 910.18 ± 45.25, 931.62 ± 46.71, and 884.16 ± 38.86 mg/g, respectively. The main fraction of MPS (MPS-2) was collected and lyophilized for further research.

### 3.5. Characterization Analysis

#### 3.5.1. Analysis of FT-IR

**Figure 4:**
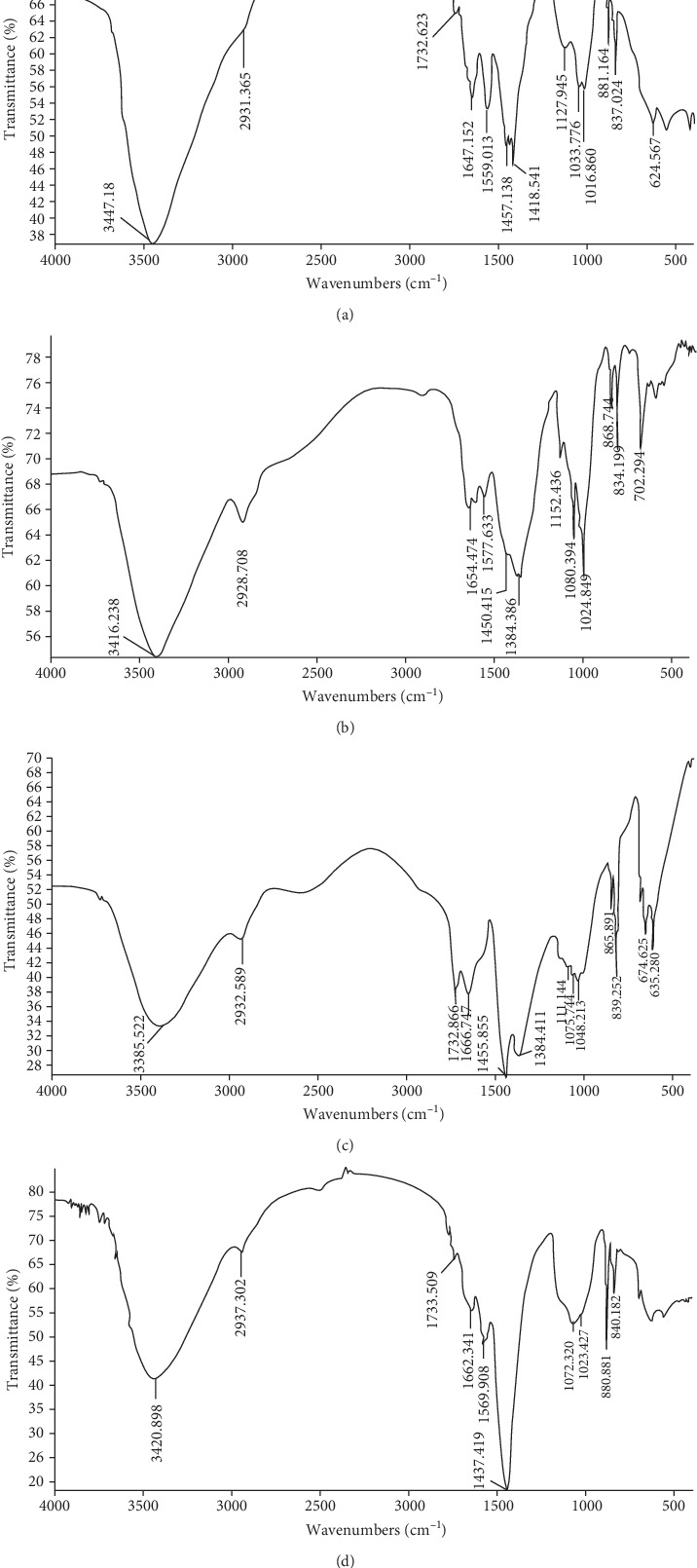
FT-IR spectra of (a) MZPS, (b) MZPS-1, (c) MZPS-2, and (d) MZPS-3.

As shown in Figure 4, a strong band between 3,600 and 3,200 cm^−1^ was attributed to the presence of O-H stretching vibration of carbohydrates. The weak band at approximately 2,900 cm^−1^ was due to C-H stretching vibration [32]. The absorption band appearing at 1,732 cm^−1^ was assigned to the C=O stretching vibration of -COOH, indicating the presence of uronic acid [33]. The absorption band at approximately 1,660 cm^−1^ was likely related to the stretching vibration of C=O. The bright absorption band at 1,200–1,000 cm^−1^ resulted from sugar ring vibrations overlapping with the C-O-C glycosidic bond vibrations and stretching vibrations of C-OH side groups [34]. The bands at approximately 891 cm^−1^ and 844 cm^−1^ indicated the existence of *β*-glycosidic bonds and *α*-glycosidic bonds, respectively [35, 36]. Therefore, MZPS and its fractions (MZPS-1, MZPS-2, and MZPS-3) showed characteristic absorption bands of polysaccharides. MZPS and its fractions had *α*-pyranose and *β*-pyranose configurations. The band at approximately 1,732 cm^−1^ indicated that MZPS, MZPS-2, and MZPS-3 contained uronic acid; MZPS-1 did not contain uronic acid.

#### 3.5.2. Analysis of Monosaccharide Composition

**Figure 5:**
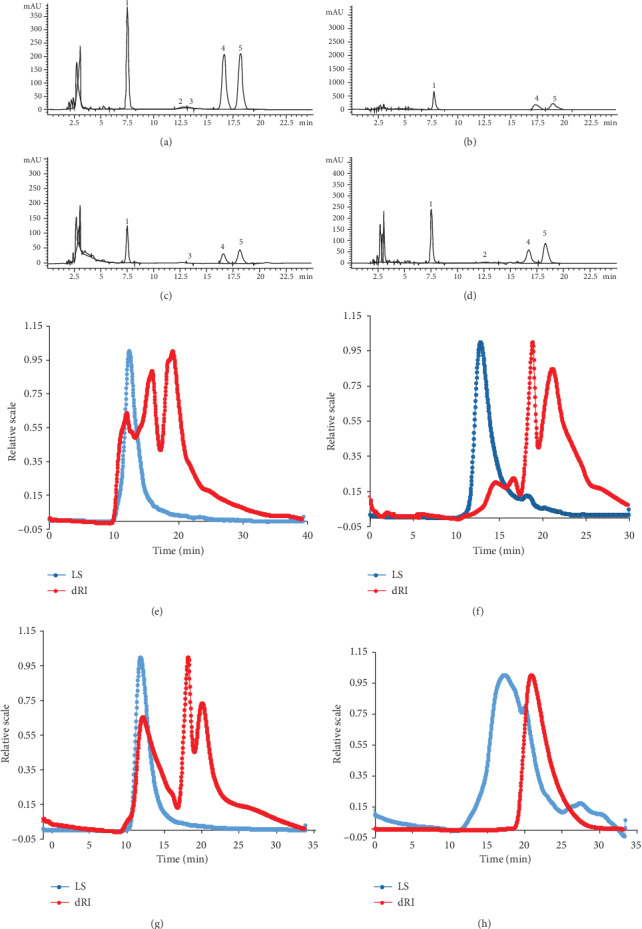
HPLC chromatograms of (a) MZPS, (b) MZPS-1, (c) MZPS-2, and (d) MZPS-3. Peaks: 1, mannose; 2, glucuronic acid; 3, galacturonic acid; 4, glucose; 5, galactose. HPGPC chromatograms of (e) MZPS, (f) MZPS-1, (g) MZPS-2, and (h) MZPS-3. LS: light scattering; dRI: differential refractive index.

As shown in Figure 5 and Table 2, galactose and glucose were the main monosaccharides in MZPS-1, while mannose was the predominant component in MZPS-2 and MZPS-3. MZPS was identified as a typically acidic heteropolysaccharide containing two uronic acids (galacturonic acid and glucuronic acid). MZPS-2 mainly contained galacturonic acid, and MZPS-3 mainly contained glucuronic acid, while MZPS-1 did not contain uronic acid (MZPS-1 was a neutral sugar eluted with deionized water), which was in agreement with the results of the FT-IR assay.

#### 3.5.3. Analysis of Molecular Weight

HPGPC chromatograms of MZPS and its fractions are shown in Figure 5. The weight-average molecular weight, number-average molecular weight, *z*-average molecular weight, and peak molecular weight of MZPS and its fractions are shown in Table 2.

#### 3.5.4. Triple Helical Structures

**Figure 6:**
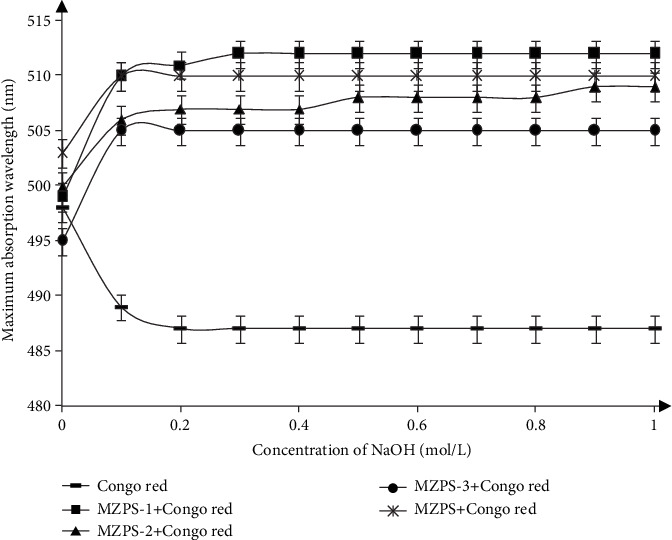
The maximum absorption wavelength of the mixture of MZPS (and its fractions) and Congo red solution at various NaOH concentrations. Data are presented as means ± SD (*n* = 3).

Congo red can form complexes with polysaccharides possessing a helical conformation, and this Congo red-polysaccharide complex could result in a bathochromic shift in the maximum absorption wavelength compared with Congo red solution. As shown in Figure 6, the maximum absorption wavelength of Congo red+MZPS (or its fractions) complex showed a distinct bathochromic shift in alkaline concentrations, compared with Congo red. Thus, it could be concluded that MZPS and its fractions (MZPS-1, MZPS-2, and MZPS-3) possessed a triple helical conformation.

### 3.6. Antioxidant Activities of Polysaccharides In Vitro

**Figure 7:**
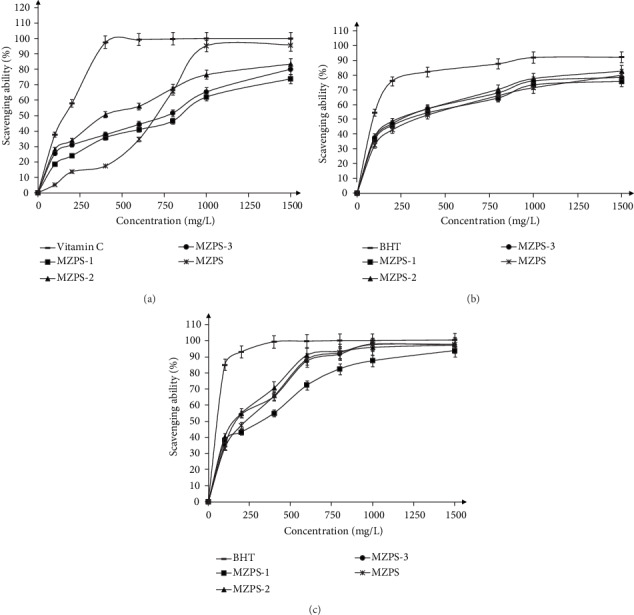
Antioxidant activities of MZPS and its fractions. (a) Hydroxyl radical-scavenging activity. (b) DPPH radical scavenging activity. (c) ABTS radical scavenging activity. Data are presented as means ± SD (*n* = 3).

Hydroxyl radicals are considered the most active and harmful free radical generated in the body. DPPH and ABTS radicals are typically used to evaluate the free radical scavenging ability of antioxidants. As shown in Figure 7, the EC_50_ values of vitamin C, MZPS, MZPS-1, MZPS-2, and MZPS-3 scavenging hydroxyl radicals were 160.65, 698.08, 845.46, 374.62, and 755.43 mg/mL, respectively. The EC_50_ values of butylated hydroxytoluene (BHT), MZPS, MZPS-1, MZPS-2, and MZPS-3 scavenging DPPH radicals were 91.34, 337.19, 294.20, 231.45, and 253.43 mg/mL, respectively. The EC_50_ values of BHT, MZPS, MZPS-1, MZPS-2, and MZPS-3 scavenging ABTS radicals were 58.84, 229.06, 315.67, 166.27, and 177.46 mg/mL, respectively. Together, the radical scavenging activity of MZPS fractions decreased in the order of MZPS-2>MZPS-3>MZPS-1.

### 3.7. Correlation of Structure and Antioxidant Activity In Vitro

These data indicated that MZPS-2 and MZPS-3 exhibited higher radical scavenging activity than MZPS-1. Compared with MZPS-1, MZPS-2 and MZPS-3 had a lower ratio of glucose in its monosaccharide composition. Moreover, both MZPS-2 and MZPS-3 contained uronic acid, but MZPS-1 did not contain. The biological activity of polysaccharides was closely related to the structural characterization. Therefore, we might rationally assume that the stronger antioxidant activity may be attributed to the presence of uronic acid and a lower proportion of glucose. These conclusions were in accordance with previous reports. For example, Liu et al. [37] reported that the acidic polysaccharides exhibited a higher radical scavenging activity than neutral polysaccharides. Xu et al. [38] found that the stronger antioxidant activity may be attributed to more uronic acid content. Gan et al. [39] found that the polysaccharide exhibiting the highest activity had a lower ratio of glucose in its monosaccharide composition and higher uronic acid content.

### 3.8. Antioxidant Activities of MZPS-2 In Vivo

**Figure 8:**
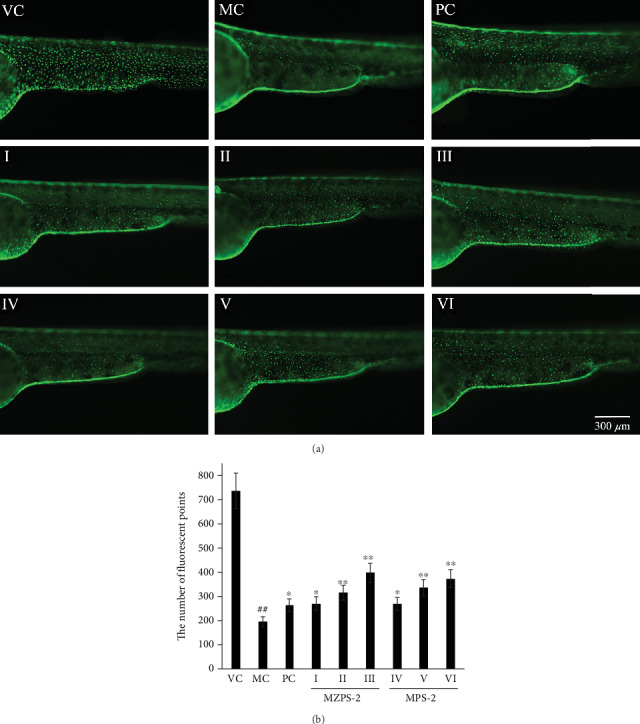
The free radical scavenging effects of MZPS-2 in zebrafish larvae. (a) Fluorescence micrographs of Tg(krt4:NTR-hKitGR)cy17 zebrafish (magnification 40x); (b) The number of fluorescent dots in zebrafish epidermal cells. VC: vehicle control group (fish water); MC: model control group (fish water containing metronidazole); PC: positive control group (vitamin C-treated group); I, II, and III: three MZPS-2-treated groups (10, 25, and 50 *μ*g/mL, respectively); IV, V, and VI: three MPS-2-treated groups (10, 25, and 50 *μ*g/mL, respectively). ^∗^*P* < 0.05, ^∗∗^*P* < 0.01 versus MC; ^##^*P* < 0.01 versus VC. Data are presented as means ± SD (*n* =10).

The transgenic zebrafish of the Tg(krt4:NTR-hKitGR)cy17 strain can be used to evaluate the antioxidant capacity of active substances. The epidermal cells of the zebrafish contain green fluorescent dots, which were constructed into a coexpression system of the specific fluorescent protein and the nitroreductase. Metronidazole can bind to nitroreductase in epidermal cells, producing an abundance of free radicals. These free radicals cause cell death and the disappearance of green fluorescent dots. Antioxidant substances can scavenge free radicals, thereby avoiding apoptosis of epidermal cells and the disappearance of green fluorescent dots.  Figure 8 illustrates that the number of fluorescent dots in the model control group (MC) was significantly lower than that in the vehicle control group (VC) (*P* < 0.01). The number of fluorescent dots was increased by MZPS-2, and MZPS-2 showed a significant concentration-dependent pattern. Compared with the MC, the number of fluorescent dots in the three MZPS-2-treated groups (I, II, and III), three MPS-2-treated groups (IV, V, and VI), and positive control group (PC) increased by 38.46%, 61.54%, 104.1%, 37.44%, 72.31%, 91.28%, and 34.36%, respectively. The ability of MZPS-2 to scavenge free radicals was not significantly different from that of MPS-2 (*P* > 0.05), indicating that the polysaccharides in MZPS-2 played a major role in the process of scavenging free radicals in the oxidative stress model in transgenic zebrafish. Zinc in MZPS-2 did not show significant antioxidant effects, which may be due to its weak scavenging effect on nitro radicals in this transgenic zebrafish model (Tg(krt4:NTR-hKitGR)cy17 strain). MZPS-2 had antioxidant capacity *in vivo*, and it might be used as a natural antioxidant to alleviate the adverse effects of oxidative stress.

### 3.9. Activities of MZPS-2 on LPS-Stimulated ROS Generation In Vivo

**Figure 9:**
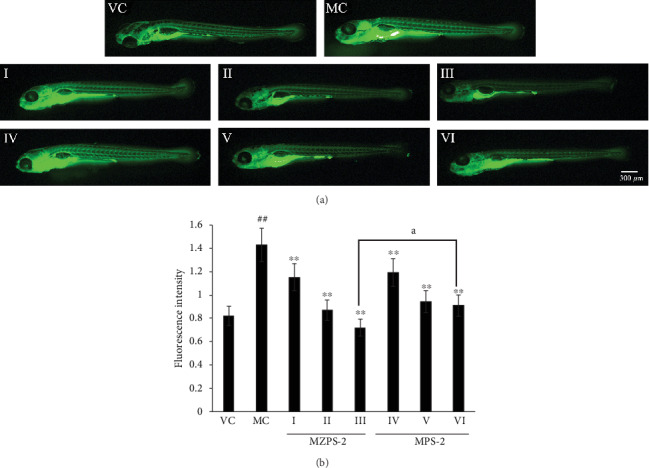
Activities of MZPS-2 on LPS-stimulated ROS generation in zebrafish larvae. (a) Fluorescence micrographs of ROS generation in zebrafish larvae (magnification 40x). (b) Quantitative analysis of ROS generation. ROS level was measured after staining with DCF-DA. VC: vehicle control group (fish water); MC: model control group (fish water containing LPS); I, II, and III: three MZPS-2-treated groups (10, 25, and 50 *μ*g/mL, respectively); IV, V, and VI: three MPS-2-treated groups (10, 25, and 50 *μ*g/mL, respectively). ^∗^*P* < 0.05, ^∗∗^*P* < 0.01 versus MC; ^##^*P* < 0.01 versus VC; ^a^*P* < 0.05 versus the other group. Data are presented as means ± SD (*n* = 10).

LPS is a peculiar substance of Gram-negative bacteria and is a triggering factor of systemic inflammatory response syndrome [40]. It can induce an inflammatory response in the body, which increases the synthesis of ROS. The overproduction of ROS not only causes oxidative stress but also is involved in the production of various proinflammatory mediators, thereby culminating in cell damage and chronic inflammation [41, 42]. The levels of ROS could be detected using an oxidation-sensitive fluorescent probe dye, 2′, 7′-dichlorodihydrofluorescein diacetate (DCF-DA). As shown in Figures 9(a) and 9(b), the fluorescence intensity was significantly increased in the MC in comparison with that of the VC, which suggests that LPS stimulation caused a significant upregulation of ROS production. However, the administration of MZPS-2 significantly reduced the level of ROS in a dose-dependent manner in LPS-stimulated zebrafish larvae. The ROS level in zebrafish larvae treated with 10, 25, and 50 *μ*g/mL MZPS-2 decreased by 19.58%, 39.16%, and 49.65%, respectively, compared to the MC. The ROS level treated with 10, 25, and 50 *μ*g/mL MPS-2 decreased by 16.78%, 34.27%, and 36.36%, respectively. Therefore, MZPS-2 can alleviate oxidative stress and inflammation by inhibiting the generation of ROS. Under high-dose conditions, MZPS-2 showed a stronger ability to inhibit ROS generation than MPS-2 (*P* < 0.05). It is possible to consider that the stronger inhibitory ability of MZPS-2 is related to the antioxidant properties of zinc. Many studies have shown that zinc can indirectly exert antioxidant effects. For instance, zinc could inhibit the generation of hydroxyl radical by antagonism with redox-active transition metals. Zinc could also induce the organism to produce metallothionein with scavenging free radical activity [43].

### 3.10. Anti-Inflammatory Activities of MZPS-2 on Neutrophil Migration In Vivo

**Figure 10:**
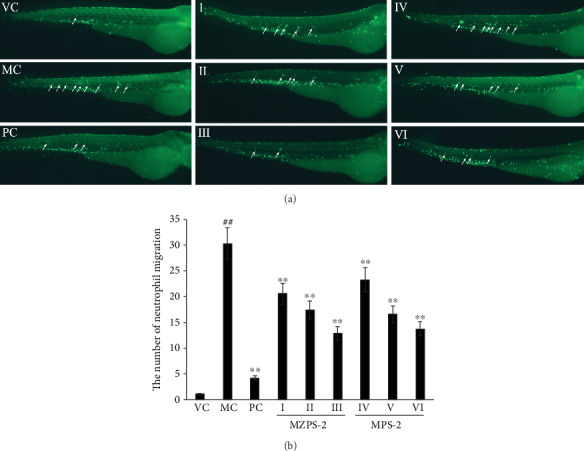
Anti-inflammatory activities of MZPS-2 on neutrophil migration in zebrafish larvae. (a) Fluorescence micrographs of neutrophil migration in Tg(Lyz:GFP) zebrafish (magnification 40x). (b) Quantitative analysis of neutrophil migration. VC: vehicle control group (fish water); MC: model control group (fish water containing CuSO_4_); PC: positive control group (indomethacin-treated group); I, II, and III: three MZPS-2-treated groups (10, 25, and 50 *μ*g/mL, respectively); IV, V, and VI: three MPS-2-treated groups (10, 25, and 50 *μ*g/mL, respectively). ^∗^*P* < 0.05, ^∗∗^*P* < 0.01 versus MC; ^##^*P* < 0.01 versus VC. The neutrophils infiltrated into the lateral line were indicated by white arrowheads. Data are presented as means ± SD (*n* = 10).

Zebrafish of the transgenic strain (Tg(Lyz:GFP) containing green fluorescent protein-labeled neutrophils allowed for quantitative visualization of an inflammatory response. When zebrafish larvae were exposed to copper sulfate, copper sulfate selectively damages the neuromasts of the lateral line system, causing migration of neutrophils toward the damaged lateral line [44, 45]. As shown in Figure 10(a), in the VC, the neutrophils are normally localized in the hematopoietic tissue in the ventral trunk, while the copper-exposed larvae (MC) showed clusters of neutrophils recruited to the lateral line.  Figure 10(b) showed a significant increase in the number of neutrophils infiltrating the lateral line in the MC compared with the VC (*P* < 0.01). However, pretreatment of larvae with MZPS-2 (I, II, and III) significantly reduced the number of neutrophils infiltrating in a dose-dependent manner. Compared with the MC, the number of neutrophils infiltrating in the MZPS-2-treated groups (I, II, and III), the MPS-2-treated groups (IV, V, and VI), and PC were decreased by 32.25%, 42.36%, 57.51%, 23.22%, 45.26%, 54.61, and 85.94%, respectively. Therefore, MZPS-2 exhibited anti-inflammatory potential *in vivo*. MZPS-2 showed similar ability to inhibit neutrophil migration as MPS-2 (*P* > 0.05), indicating that the polysaccharide in MZPS-2 played a major role in inhibiting neutrophil migration in the transgenic zebrafish inflammation model.

## 4. Conclusions

*T. ganbajun* had a strong ability to absorb and transform zinc. When *T. ganbajun* was grown under zinc homeostasis conditions, zinc can be compartmentally stored in small vesicles and accumulated in the form of zinc-enriched polysaccharides. Zinc polysaccharides are an ideal bioorganic zinc, which has the biological functions of organic zinc and polysaccharides. In the present study, MZPS and its three major fractions (MZPS-1, MZPS-2, and MZPS-3) were successfully extracted and purified. The zinc content of MZPS and MZPS-2 was 25.0 ± 1.27 and 25.9 ± 1.33 mg/g, respectively. For antioxidant testing *in vitro*, MZPS and its fractions had considerable antioxidant activity. Comparative analysis of structural features and *in vitro* antioxidant activity revealed that higher activity might be attributed to a lower ratio of glucose in the monosaccharide composition and the presence of uronic acid. For antioxidant testing *in vivo*, we found that MZPS-2 had a remarkable ability to scavenge free radicals (*P* < 0.01). For anti-inflammation testing *in vivo*, the results showed that MZPS-2 could inhibit the generation of ROS caused by inflammation (*P* < 0.01) and the recruitment of neutrophils toward the injury site (*P* < 0.01). Therefore, MZPS-2 could potentially be used as a new zinc supplement, which might have a beneficial effect during the treatment of inflammation or oxidative stress caused by zinc deficiency.

## Figures and Tables

**Table 1:** Zinc enrichment ratio of cellular components.

	Proteins	Polysaccharides	Nucleic acids	Mycelia
Yield (mg/g)	532.25 ± 21.52	94.00 ± 3.5	6.15 ± 0.27	/
Zinc content (mg/g)	0.393 ± 0.01	24.40 ± 1.05	0.552 ± 0.02	10.7 ± 0.46
Zinc enrichment ratio (%)	1.95	21.44	0.032	/

Yield (mg/g) = weight of each component (mg)/weight of mycelia powder (g); zinc enrichment ratio (%) = zinc content of each component (mg/g) × yield (g/g)/zinc content of mycelia powder (mg/g).

**Table 2:** Monosaccharide composition and molecular weight of MZPS and its fractions.

	MZPS	MZPS-1	MZPS-2	MZPS-3
Monosaccharide composition (mol%)				
Mannose	25.05	3.57	40.65	42.33
Glucuronic acid	0.97	/	/	3.71
Galacturonic acid	0.24	/	0.66	/
Glucose	43.07	46.54	26.35	19.53
Galactose	30.66	49.89	32.33	34.42
Molecular weight (Da)				
Mw	5.592 × 10^5^	4.145 × 10^5^	5.118 × 10^5^	5.831 × 10^3^
Mn	2.317 × 10^5^	8.367 × 10^4^	1.097 × 10^4^	1.513 × 10^3^
Mz	1.153 × 10^6^	6.015 × 10^6^	1.492 × 10^6^	1.131 × 10^6^
Mp	2.035 × 10^5^	6.488 × 10^4^	4.846 × 10^4^	2.222 × 10^3^

Mw: weight-average molecular weight; Mn: number-average molecular weight; Mz: z-average molecular weight; Mp: peak molecular weight.

## Data Availability

The data used to support the findings of this study are available from the corresponding authors upon request.

## Abbreviations

MZPS: Mycelia zinc polysaccharides
MPS: Mycelia polysaccharides
FT-IR: Fourier transform infrared spectroscopy
HPLC: High-performance liquid chromatography
HPGPC: High-performance liquid gel permeation chromatography
DPPH: 2, 2-diphenyl-1-picryl-hydrazyl
ABTS: 2, 2′-azino-bis (3-ethylbenzothiazoline-6-sulfonic acid)
BHT: Butylated hydroxytoluene
Hpf: Hours post fertilization
MC: Model control group
VC: Vehicle control group
PC: Positive control group
LPS: Lipopolysaccharide
ROS: Reactive oxygen species
DCF-DA: 2′, 7′-dichlorodihydrofluorescein diacetate.
